# Clinical application and efficacy analysis of partial cystectomy combined with intravesical chemotherapy in muscle-invasive bladder cancer

**DOI:** 10.1186/s12894-023-01267-w

**Published:** 2023-05-11

**Authors:** Bin Zhang, Tengfei Liu, Yang He, Dali Han, Peng Qi, Duo Zheng, Junyao Liu, Xingxing Zhang, Zhongjin Yue, Panfeng Shang

**Affiliations:** 1 Department of Urology, Institute of Urology, Gansu Nephro-Urological Clinical Center, Key Laboratory of Urological Diseases in Gansu Province, Lanzhou University Second Hospital, Lanzhou, 730030 Gansu China; 2 Department of Urology, General Hospital of Pingmei Shenma Group, Pingdingshan, 467002 Henan China

**Keywords:** Muscle-invasive bladder cancer, Partial cystectomy, Radical

## Abstract

**Objectives:**

Comparing the long-term tumor control results of partial cystectomy(PC)and radical cystectomy(RC)in the treatment of muscle-invasive bladder cancer, and to explore the feasible method of bladder preservation therapy (BPT)in patients with MIBC.

**Methods:**

We retrospectively analyzed the clinical data of 102 patients with muscle-invasive bladder cancer in our hospital between January 2012 and December 2018, of whom 32 cases in the partial cystectomy group and 70 cases in the radical cystectomy group. We performed a comparative analysis of patient general information, perioperative-related indicators and postoperative follow-up data, comparing OS, PFS, and DSS at 1, 2, 3, 4, and 5 years in both groups, and comparing tumour recurrence and metastasis in postoperative patients.

**Results:**

All the 102 cases in this study were successfully completed. Partial cystectomy group and Radical cystectomy group median operating time (169.50(130.00 ~ 225.25) min and 420.00(343.75 ~ 483.75) min, p < 0.001), median intraoperative blood loss was (100(50 ~ 100)ml and 400(200 ~ 1000)ml, p < 0.001), median perioperative blood transfusion volume (0(0 ~ 0)ml and 600(150.00 ~ 906.25)ml, p < 0.001), median total hospital stay (18(14.25 ~ 20.00) and 24.5(20.00 ~ 34.25) days, p < 0.001), median preoperative preparation time (7(4.25 ~ 8.00) and 10(8.00 ~ 13.00) days, p < 0.001), median postoperative hospital stay (9(8.00 ~ 13.50) and 14(11.00 ~ 21.25) days, p < 0.001), the incidence of perioperative blood transfusion was (15.6% and 75.7%, p < 0.001), the incidence of surgical complications was(28.1%(9/32) and 50.0%(35/70), p = 0.033), average hospitalization cost ((26435.76 ± 9877.82) yuan and (58464.36 ± 19753.13) yuan, p < 0.001), the differences were statistically significant (p < 0.05). Perioperative mortality (0 vs. 2.9%(2/70), p = 1), and OS at 1, 2, 3, 4, and 5 years after surgery were (80.0%, 59.8%, 56.1%, 51.0%, 44.6% vs. 76.5%, 67.4%, 64.9%, 57.9%, 52.6%, p = 0.524), PFS (68.2%, 64.6%, 60.3%, 54.8%, 54.8% vs. 82.7%, 78.3%, 75.4%, 67.3%, 62.1%, p = 0.259). DSS (89.9%, 72.4%, 68.6%, 68.6%, 62.4% vs. 87.3%, 83.4%, 80.9%, 73.6%, 68.0%, p = 0.424), and the incidence of tumor recurrence or metastasis was (40.0%(12/30) vs. 25.4%(16/63), p = 0.151), the differences were not statistically significant (p > 0.05).

**Conclusion:**

In patients with limited solitary T2N0M0 and T3N0M0 muscle-invasive bladder cancer, partial cystectomy plus bladder instillations treatment can achieve comparable tumour control to radical cystectomy. However, patients in the PC group have significant advantages in terms of operative time, intraoperative bleeding, intraoperative and postoperative blood transfusion, preoperative preparation time, total hospital stay, postoperative recovery time, operative costs and operative complications. This option may be considered for such patients with a need for bladder preservation.

## Introduction

Bladder cancer is a common malignant tumor in the urinary and male reproductive system, seriously endangering human health, about 30% of the first diagnosis is muscle-invasive bladder cancer (MIBC) [1]. Radical cystectomy (RC) + pelvic lymph node cleaning + urinary diversion has long been regarded as the standard treatment for MIBC, and its local tumor control effect is supported by a lot of experimental data [2, 3]. However, due to the complicated operation and long time, large trauma, large changes for normal physiological structure, poor postoperative quality of life, and mostly elderly patients, RC is closely related to postoperative complications, postoperative complications and mortality rate can reach 31 -51% and 1.5 -2.7% [4–6]. Therefore, some patients with MIBC refused a radical cystectomy, bladder preserving therapy(BPT)is feasible in partial local individual-onset patients.

Current guidelines support the use of triple (TURBT + radiotherapy + chemotherapy) therapy as an alternative to RC, but emphasise the need for appropriate patient selection, close multidisciplinary collaboration between surgeons, radiologists and oncologists, and long-term monitoring, so the chances of recurrence remain high [7, 8]. Triple therapy is not suitable for all patients, and there is no uniform standard for screening patients with MIBC before receiving triple therapy at this stage.

Partial cystectomy as a surgical procedure that both preserves bladder function and completely removes the lesion. It has been widely used in the treatment of MIBC since the mid-20th century. However, due to the lack of strict patient screening and different surgeon levels, the survival rate of patients at 5 years after surgery was low and the postoperative recurrence rate was high [9]. As RC has gradually become the standard of care for MIBC, the use of PC in the clinic has gradually decreased. In recent years, as patients have become more demanding in terms of quality of life, partial cystectomy, which allows for complete resection of the tumour and preservation of the bladder, has received renewed interest from surgeons. Therefore, in clinical work could partial cystectomy combined with bladder instillations be an option for patients with partial muscle-invasive bladder cancer to achieve comparable outcomes to radical treatment. PC has the advantages of minimal surgical trauma, no need for urinary diversion and preservation of sexual function, all of which are prerequisites for improving patients’ quality of life. In addition, combined with intravesical irrigation therapy has significant efficacy in reducing tumour recurrence. We retrospective analyzed the clinical data of some patients with PC and RC from January 2012 to December 2018 with MIBC and conducted follow-up, to explore whether the treatment of PC + bladder instillations can achieve the same tumor control results in strictly selected MIBC patients as RC.

## Patients and methods

### General Information

We retrospectively collected patients diagnosed with muscle-invasive bladder cancer in the Urology Department of Lanzhou University Second Hospital between January 2012 and December 2018. This study received the Lanzhou University Second Hospital Ethical Committee approval(2017 A-053). Patient inclusion criteria were: (1) initial diagnosis of patients with muscle-invasive bladder cancer; (2) patients with pathological stage T2-T3 without lymph node metastases and distant metastases; (3) Patients with muscle-invasive bladder cancer with localized solitary origin and less than 2 lesions. (4) postoperative pathology suggestive of uroepithelial carcinoma;(5)patients with complete case information. Exclusion criteria: (1) patients with recurrent muscle-invasive bladder cancer; (2) tumours near the neck of the bladder; (3) patients with small volume bladders; (4) patients who have had radiotherapy to the lower abdomen; (5)Patients with muscle-invasive bladder cancer with high risk of recurrence after comprehensive evaluation.

### Treatment options

#### PC group

The patients in this group underwent PC (open and laparoscopic surgery) and immediate and maintenance bladder irrigation with epirubicin or hydroxycamptothecin after surgery. Open surgery is performed through a median or curved incision in the abdomen. A 2 cm skin incision is made below the umbilicus, a 10 mm Trocar is placed, the pneumoperitoneum is connected and the pressure is controlled at 12–15 mmHg, the laparoscope is placed, a 10 mm and a 5 mm Trocar are placed on both sides of the external border of the rectus abdominis muscle under direct vision and the corresponding laparoscopic instruments are placed. The tumour is removed together with 2 cm of surrounding normal tissue. If the resection includes the uretero-vesical junction on one side, a simultaneous uretero-vesical reimplantation with a double J-tube should be performed. Intraoperative surgery, the internal pelvic lymph nodes, including the internal iliac, external iliac lymph nodes, and surrounding the obturator nerve, were explored, combined with preoperative imaging examination, and the suspicious enlarged lymph nodes were cleaned and sent for pathological examination.

In this group, conventional bladder instillations therapy was administered 4 weeks after surgery with pirorubicin or hydroxycamptothecin. Post-operative bladder instillations is 1 time per week for 8 times and then 1 time per month for 12 times. Pirorubicin was 30 mg for every time and hydroxycamptothecin was 20 mg for every time.

#### RC group

Patients in this group underwent RC + urinary flow diversion + pelvic lymph node dissection with no other adjuvant treatment. For open surgery, the median lower abdominal incision of about 15 cm long to the upper of the navel was taken. For laparoscopic surgery, 1 cm incision was taken from the upper of the navel, pneumoperitoneal needle puncture and CO_2_ injection, abdominal pneumoperitoneum pressure to 12 ~ 15mmHg, puncture 10 mmTrocar, puncture 10 mmTrocar in the left and right inferior umbilical rectus ventral muscle respectively, puncture 5 mmTrocar in the right and left anterior superior iliac spine 2 ~ 3 cm above and medial respectively, and the corresponding laparoscopic surgical instruments were inserted. Radical resection of the bladder and surrounding adipose tissue, distal ureter, men including the bladder, prostate, seminal vesicle, women underwent anterior pelvic visceral enucleation, including the bladder, uterus, fallopian tubes, ovaries. Part of the urethra and the anterior wall of the vagina are removed when infiltration into the bladder triangle or posterior urethra [10, 11]. All patients underwent standard pelvic lymph node dissection, including the removal of the extrailiac vessels and internal iliac vessels around the common iliac vessels, and the details can be reported in the earlier literature of our hospital [12]. The laparoscopic group used a median 10 cm longitudinal incision for urinary flow diversion or reconstruction.

### Follow-up

For the patients finally included in this study, we will check the regular return visit records or ask the patient or their family members about the review results in other hospital. The current survival status, quality of life and tumor control of the patients were clarified, and the cause of death and survival time were clarified for the patients who died.

### Statistical analysis

The data were processed using the SPSS 25.0 statistical software. Measurement data conforming to normal distribution were represented by Mean ± SD and t-test for comparison between groups;skewed distribution measurement data were represented using median and interquartile spacing, and compared between groups using the Mann-Whitney U test;count data were expressed as frequency or percentage, and groups were compared using the X² test or Wilcoxon rank sum test. Disease-specific survival (DSS), progression-free survival (PFS), and overall survival (OS) were calculated using the Kaplan-Meier method. The difference of p < 0.05 was statistically significant.

## Results

### Baseline patient data for two groups

A total of 102 patients included in this study, of which 82 were male and 20 were female, with a male to female ratio of 4.1:1, aged 28–89 years, median age 65 (56.0–72.0) years. The PC group consisted of 32 cases, 27 males and 5 females, male to female ratio 5.4:1, age 43–85 years, median age 65.5 (58.5–73.5) years.17 patients (53.1%) with underlying disease. The RC group consisted of 70 patients, 55 males and 15 females; the male to female ratio was 3.7:1, aged 28–89 years;the median age was 65 (55.0–72.0) years. There were no significant differences in gender, age, TNM stage of tumor, basic disease in the two groups (P > 0.05). Specific general information is shown in Table 1.

**Table 1:** General information table

	PC group	RC group	P value
**Gender**			
Male	27(84.4)	55(78.6%)	0.493
Female	5(15.6)	15(21.4%)	
**Age**	65.5(58.5–73.5)	65.0(55.0–72.0)	0.564
**Basic disease**	17(53.1%)	26(37.1%)	0.129
**Characteristics of surgical specimens**			
Histological variation(%)			
Squamous differentiation	2(6.2%)	5(7.1%)	0.761
Adenal differentiation	0	1(1.4%)	
Giant cell type	0	0	
Lymphomatoid and plasmacytoid changes	0	1(1.4%)	
intravascular tumour embolism or invasion	7(21.9)	28(40.0%)	0.074
Nerve invasion	2(6.2)	11(15.7)	0.220
**Pathological stage**			
T2N0M0	29(90.6)	65(92.9)	0.703
T3NOMO	3(9.4)	5(7.1)	

### Perioperative condition comparison between the two groups

Mean operation time between the PC and RC groups was [169.50(130.00 ~ 225.25)min and 420.00(343.75 ~ 483.75)min, p<0.001], Average hospitalization costs was [(26435.76 ± 9877.82) yuan and (58464.36 ± 19753.13) yuan, p < 0.001], The median intraoperative blood loss was measured between [100(50 ~ 100)ml and 400(200 ~ 1000)ml, p<0.001], The median perioperative blood transfusion volume was [0(0 ~ 0)ml and 600(150.00 ~ 906.25)ml, p < 0.001], The median total length of stay was [18(14.25 ~ 20.00)days and 24.5(20.00 ~ 34.25) days, p < 0.001], The median postoperative length of stay was measured between [9(8.00 ~ 13.50)days and 14(11.00 ~ 21.25)days, p < 0.001], The median preoperative preparation time was [7(4.25 ~ 8.00)days and 10(8.00 ~ 13.00)days, p < 0.001], The incidence of perioperative blood transfusions was [15.6% and 75.7%, p < 0.001], the differences were all statistically significant (p < 0.05). There was no significant difference in the perioperative mortality rates [0 and 2.9%, p = 1, and p > 0.05]. Perioperative period data is shown in Table 2.

**Table 2:** Perioperative period data

	PC group	RC group	*P value*
Operation time(min)	169.50(130.00~225.25)	420.00(343.75~483.75)	P<0.001
Intraoperative bleeding(ml)	100(50~100)	400(200~1000)	P<0.001
Blood transfusion quantity(ml)	0(0~0)	600(150.00~906.25)	P<0.001
Blood transfusion treatment(%)	5(15.6%)	53(75.7%)	P<0.001
Total length of stay(day)	18(14.25~20.00)	24.5(20.00~34.25)	P<0.001
Pre-operative hospital stay(day)	7(4.25~8.00)	10(8.00~13.00)	P<0.001
Postoperative hospital stay(day)	9(8.00~13.50)	14(11.00~21.25)	P<0.001
Surgical complications(%)	28.1%(9/32)	50.0%(35/70)	0.033
30-day complication rate, (%)			
surgical site infections	9.4%(3/32)	10%(7/70)	1.000
Perioperative deaths(%)	0	2(2.9%)	1.000
Recurrence or distant metastasis(%)	40.0%(12/30)	25.4%(16/63)	0.151
Bladder cancer-specific death(%)	33.3%(10/30)	22.2%(14/63)	0.219

### Postoperative bladder perfusion in PC patients

In the PC group, four postoperative patients underwent hydroxycamptothecin bladder instillations therapy, the remaining 28 patients were given pirorubicin bladder instillations chemotherapy.

### Comparison of the surgical specimens between the two groups

There were 7 (21.9%), 2 (6.1%) and 1 (3%) cases of intravascular tumour embolism or invasion, nerve invasion and histological variation respectively in the pathological specimens of the PC group; there were no cases of multiple tumours, 29 (90.7%) in the T2N0M0 stage and 3 (9.3%) in the T3N0M0 stage. Among the pathological specimens in the RC group, there were 28 (40.0%), 11 (15.7%) and 7 (10.0%) of intravascular tumour embolism or invasion, nerve invasion and histological variation respectively; there were no cases of multiple tumours, 65 (92.9%) had stage T2N0M0 and 5 (7.1%) had stage T3N0M0. There were no significant differences in TNM stage, intravascular tumour embolism or invasion, nerve invasion, and histological variation (p > 0.05). See Table 1 for details.

### Comparison of postoperative follow-up results in the two groups

**Fig. 1:**
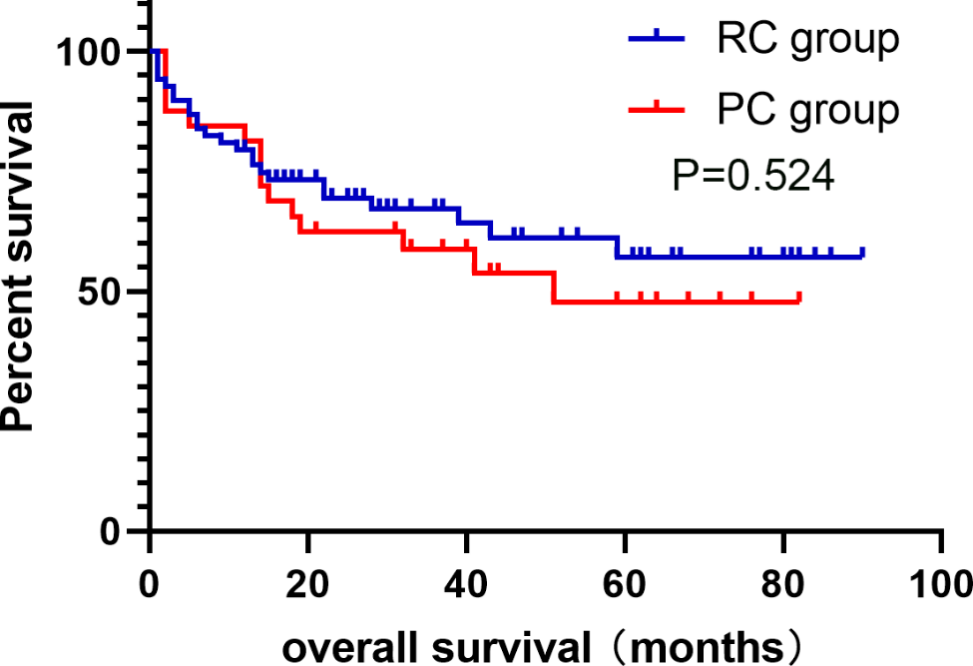
Kaplan-Meier curves of overall survival probability in patients who received PC and RC

**Fig. 2:**
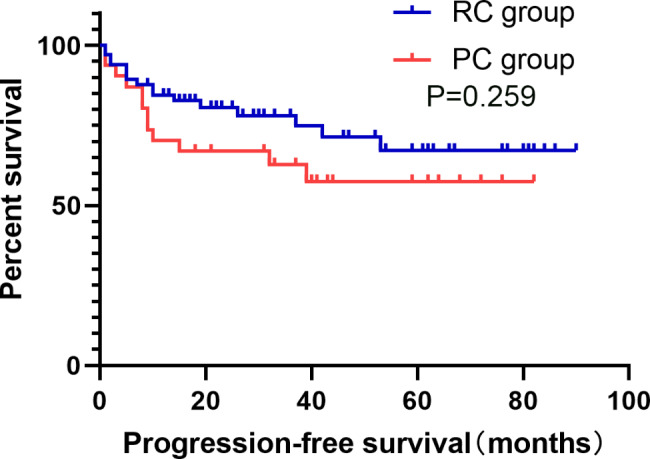
Kaplan-Meier curves of progression-free survival probability in patients who received PC and RC

**Fig. 3:**
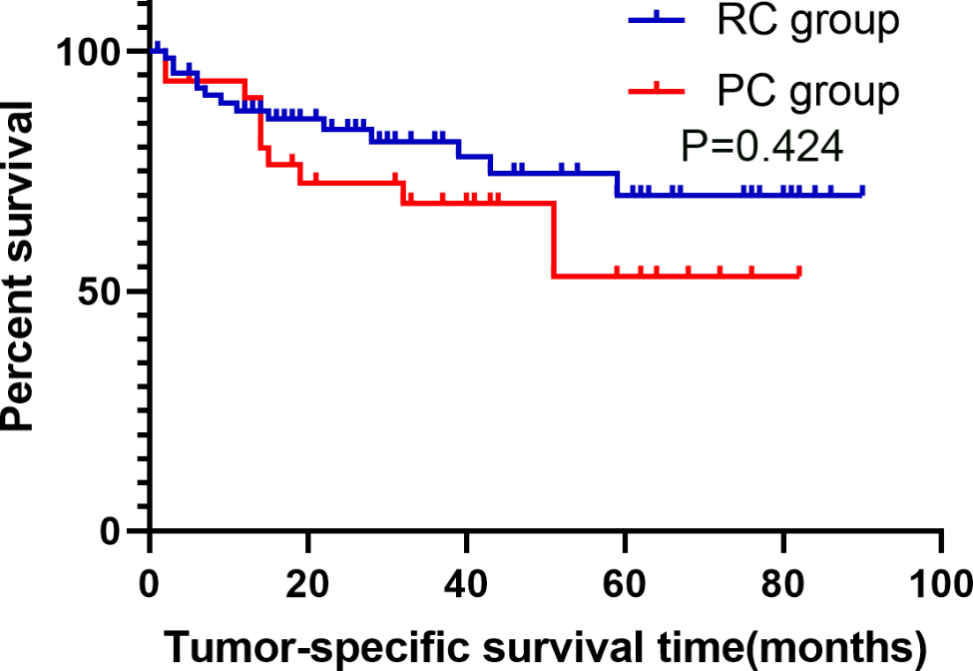
Kaplan-Meier curves of disease specific survival probability in patients who received PC and RC

The PC and RC groups have 2 cases and 6 cases patients lost follow-up, 6.3% and 8.6%, respectively. The overall loss to follow-up rate was 7.8%. The PC group with a median follow-up of 32 (14 to 51) months, and the RC group with a median follow-up of 22.5 (12.25 to 45.25) months. OS at 1, 2, 3, 4 and 5 years after surgery in the PC and RC groups were [80.0%、59.8%、56.1%、51.0%、44.6% and 76.5%、67.4%、64.9%、57.9%、52.6%, p = 0.524]; PFS were [68.2%、64.6%、60.3%、 54.8%、54.8% and 82.7%、78.3%、75.4%、67.3%、62.1%, p = 0.259]; DSS was [89.9%、72.4%、68.6%、68.6%、62.4% and 87.3%、83.4%、80.9%、73.6%、68.0%, p = 0.424], respectively, and the incidence of tumour recurrence or metastasis were [40%(12/30) and 25.4%(16/63), p = 0.151], respectively, and none of the differences were statistically significant (p > 0.05) (for details, see Figs. 1, 2 and 3). The incidence of surgical complications in the PC and RC groups were [28.1%(9/32) and 50.0%(35/70), p = 0.033], respectively, with a statistically significant difference (p < 0.05). Two patients in the PC group presented with intravesical recurrence with muscle-invasive at 26 and 9 months postoper atively, respectively, and were treated with salvage RC, one patient was treated with postoperative adjuvant chemotherapy with GC regimen, during which urethral metastases were detected and urethrectomy was performed, and he is still alive, another patient died of systemic multiple metastases more than 6 months postoperatively without postoperative adjuvant therapy. Two patients presented with intravesical recurrence without infiltration at 9 and 3 months postoperatively, respectively, and were treated with TURBT, one case died 42 months after surgery due to pancreaticobiliary metastases and one case developed a recurrence shortly after surgery and eventually died at one month after surgery due to pulmonary embolism. The bladder preservation survival rate in the PC group was 43.8%. The bladder preservation survival rate of all surviving patients was 93.3%.

## Discussion

Bladder cancer is prone to multiple occurrences, recurrence and metastases, and different patients have different risks of progression, so bladder preservation therapy cannot be applied to all MIBC patients and strict patient selection is required before surgery. Moreover, Some patients with uroepithelial carcinoma of the bladder can be treated with PC to achieve the same local treatment effect as RC with complete tumour removal and lymph node dissection [13–15]. In clinical practice, patients with MIBC are often divided into medium-risk and high-risk groups based on certain risk factors (MIBC is a malignant tumour of the urinary tract and has a greater risk, so some experts believe that there is no low-risk group) [16, 17]. The risk of tumour progression and metastasis after surgery is much greater in the high-risk group than in the medium-risk group. In order to demonstrate the effectiveness of PC + bladder instillations treatment, the patients enrolled in the PC group were strictly selected in this study. The main criteria were primary tumour, single tumour, tumour TNM stage within T2-3N0M0 and pathological type of uroepithelial carcinoma. A total of 102 patients, 32 in the PC group and 70 in the RC group, were enrolled in the study according to strict enrolment criteria. In the PC group, bladder instillations was routinely performed 2–4 weeks after surgery. There were no statistically significant differences between the two groups in terms of age, gender, tumour stage, presence of vascular and nerve invasion, presence of carcinoma in situ and presence of tissue variation. Both groups had PC group superior to RC group in postoperative complications, but there was no significant difference in infection of surgical incision within 30 days after surgery. Some studies have shown that the infection of the surgical site is closely related with age, BMI, ASA score, having diabetes and other underlying diseases [18]. The occurrence of postoperative complications is also closely related to the surgical method. For patients undergoing laparoscopic surgery, the incidence of postoperative complications is lower and the postoperative recovery is faster [19].

The follow-up results showed that the 5-year OS, PFS and DSS in the PC group were 44.6%, 54.8% and 62.4%, respectively, while the 5-year OS, PFS and DSS in the RC group were 52.6%, 62.1% and 68.0%, respectively, there is no statistically significant difference between the two groups. The combined treatment regimen can achieve the same tumour control effect as RC treatment. Fujii et al. [20] performed PC-based BPT on 84 MIBC patients in 2015, and the long-term postoperative follow-up showed a high 5-year DSS and OS of 96% and 94%, which was significantly different from the results of our study ( PC group DSS and OS of 62.4% and 44.6%, respectively). Fujii et al. initially enrolled a total of 292 patients with MIBC, first undergoing tumour reduction surgery by TURBT, followed by low-dose concurrent radiotherapy for the patients, finally, partial bladder resection was performed in 84 patients with non-muscle invasive bladder cancer residuals that were isolated and small in size, did not invade the bladder neck, did not have multiple carcinomas in situ, and did not have residual cancer tissue at the surgical site after radiotherapy the regimen involved radiotherapy and theoretically had a therapeutic effect on the possible presence of micrometastases, and the regimen was preceded by TURBT and the results of the procedure were assessed prior to PC, theoretically excluding patients with MIBC who were at greater risk of progression already. This shows that radiotherapy can control tumors to some extent and can greatly improve DSS and OS in patients. In recent years, radiotherapy has become a more attractive alternative to metastatic lesion clearance in oligometastatic patients because it is less invasive and can significantly improve patient outcomes [21]. Furthermore, Chalasani V [22] has stated that the presence of tissue variants in pathological specimens should be taken seriously as they may indicate the risk of disease progression, and in this study there were 2(6.1%) and 7 (10%) cases in each of the PC and RC groups (p = 0.716) with no statistically significant difference between the two groups, but the presence of tissue variants may have reduced both groups due to respective OS and DSS, and these may have caused these differences. The incidence of surgical complications in the PC and RC groups was 28.1% (9/32)) and 50.0% (35/70) respectively (p = 0.033), a statistically significant difference (p < 0.05), with the RC group having a higher rate of postoperative complications, a result that is in line with the findings of several other studies [23, 24].

Both groups had a higher risk of tumour recurrence and metastasis after surgery. A total of 40% (12/30) of the PC group had recurrence or metastasis. There were 6 patients with distant metastases, including 1 small bowel metastasis who died more than 2 months after reduction surgery, 1 lung metastasis, 1 pharyngeal metastasis and 3 lymph node metastases, for which no special treatment was given. Six patients had local recurrences, two of which had muscular infiltration and were treated with salvage RC, one of whom died of multiple metastases more than 6 months after surgery, and one of whom was treated with postoperative gemcitabine plus cisplatin (GC) chemotherapy and survived despite urethral metastases, as the tumour was controlled by timely detection and complete excision. Two patients were treated with TURBT after postoperative detection of recurrence in the bladder, one died shortly after recurrence, one eventually developed pancreaticobiliary cancer and died, and the other two patients died after recurrence without special management. As the study was retrospective, there was not complete uniformity in terms of treatment strategy, while some patients and families refused radical bladder cancer surgery and failed to achieve timely salvage RC treatment for patients with recurrence in the bladder detected early as recommended by the guidelines [25], which may have reduced the OS to some extent for the PC group. A total of 25.4% (16/63) of the patients in the RC group had metastases after surgery, including 3 urethral metastases, 4 colon metastases, 3 liver metastases, 1 lung metastasis, 2 bone metastases, 4 single site lymph node metastases and 6 multiple metastases (lung, colorectal, lymph node and bone). 3 patients with urethral metastases underwent urethrectomy and 1 is currently alive; 2 patients with lymphatic metastases were treated with local radiotherapy and 1 is still alive. The remaining patients with metastases were not given any special treatment and all had died by the end of the follow-up period. Therefore, it is extremely important for patients with MIBC to have regular post-operative reviews to detect metastases or recurrence early and to intervene in appropriate ways (e.g. salvage RC, radiotherapy, chemotherapy, local tumour reduction) to prolong their survival if the tumour can be controlled. In this study BPT achieved similar tumour control to RC, with a bladder preservation survival rate of 45.5% and an even higher bladder preservation rate of 93.8% in surviving patients, we believe that the BPT option is worth exploring.

In this study, we found that patients with partial bladder resection were not statistically significant in tumor recurrence or distant metastasis and bladder cancer-specific death, which may not have a longer follow-up time, with some limitations, and a longer follow-up time is needed to clarify the clinical efficacy of partial bladder resection. In addition, considering that this study is a retrospective single-centre study, the small sample size affects the reliability of the evidence and a multi-centre, large sample size, randomised controlled study could be conducted subsequently to verify the feasibility of this treatment option.

## Conclusions

In patients with limited solitary T2N0M0 and T3N0M0 muscle-invasive bladder cancer, partial cystectomy plus bladder instillations treatment can achieve comparable tumour control to radical cystectomy. In our study, patients in the PC group have significant advantages in terms of operative time, intraoperative bleeding, intraoperative and postoperative blood transfusion, preoperative preparation time, total hospital stay, postoperative recovery time, operative costs and operative complications. This option may be considered for such patients with a need for bladder preservation.

## Data Availability

Raw data may be requested from the corresponding author with the permission of the institution.
